# Should Panel Doctors Be Eligible for Honorary Staff Appointments?

**Published:** 1913-05-10

**Authors:** 


### SHOULD PANEL DOCTORS BE ELIGIBLE FOR HONORARY STAFF APPOINTMENTS?

### The Duties of the Two Posts Compared.

BY AN HONORARY MEDICAL OFFICER.

This is not a political question. The medical staffs of our large hospitals will answer it with an emphatic "No." But they alone are not the governors of our institutions, therefore it is necessary to discuss the subject more fully.

This question has arisen in the Royal Infirmary, Sunderland. Hitherto the staff have been prohibited from taking appointments under the Poor Law, provident dispensaries, and clubs. The opinion has been expressed that work under the National Insurance Act is not "club" work; though by most people this opinion will not be shared. It is agreed to alter the rule in order that no doubt may be left on the point. The staff consists of five honorary physicians, five honorary surgeons, and six assistants. Only one of the senior staff and two of the assistants are "on the panel." Two of the seniors (including the one previously mentioned) have partners doing panel work.

It is necessary for the governors to consider:—(1) What is the nature of the duties of the physicians and surgeons elected on their staffs; and (2) What class of medical men should they be selected from in order that the patients of their institution should receive the benefit of the highest skill their town affords. For, if the present members of hospital staffs be allowed to undertake work under the National Insurance Act, it is plain that "panel" doctors will form in the future one class of medical men from whom the physicians and surgeons of our hospitals, at any rate in the provinces, may be selected—a more serious consideration, for obvious reasons, than that of the present staffs undertaking panel work.

The plain duty of committees is to elect men whose natural ability and previous training best fits them to undertake the often difficult and arduous duties of hospital work. No other consideration must be allowed to influence them in recording their votes; otherwise they fail in their plain duty to the hospital. Whether a committee of laymen are the best judges of the capabilities of applicants for these posts need not be discussed here. What, then, are the duties of a member of the staff of a hospital? As an example, those of a surgeon will be given:—

### A Hospital Surgeon's Duties.

1. By previous work and innate capacity he must be well fitted to undertake numerous, difficult operations of almost daily occurrence, which frequently incur laborious work, involving both physical and mental strain for several hours at a stretch. The science and art of surgery is going ahead, and has gone ahead by vast strides, especially during the last ten years, and now operations have been increased greatly not only in number but in extent and complexity.

2. He must spend much time in study and in keeping himself abreast of the current literature of his art, of this country and of America at least.

3. He must visit the operating theatres of other surgeons in this country, and if possible some of those abroad.

From what class of medical men should such an officer be selected? Medical men may now be classified thus, remembering that members of each class frequently do work in the other classes:—

Consulting physicians and surgeons, who are few in the provinces, and are mostly to be found in the big towns where there are medical schools; private medical practitioners, a class in which some from special previous appointments, inclination, and study are fitted or are fitting themselves to become physicians, surgeons, or specialists in some one branch of medicine or surgery. Lastly, there are panel doctors. The following particulars will help to illustrate their work.

The Borough of Sunderland has a population of about 154,000, of whom 40,000 are insured persons. Each insured person has probably two others dependent upon him; thus 120,000, or more than three-fourths of the population, belong to the insured class. There are forty-two doctors on the panel; twenty-four, who are practising, are not on the panel; and about twelve others are mainly officials. Taking the insured class as 120,000, and assuming they will all be attended by panel doctors, each panel doctor will have an average of 2,850 possible patients—more than double the average persons (1,400) per doctor in Great Britain; some men have over 2,000 "insured" persons on their panel. It is well known that contract and "club" patients give about three times as much trouble and work as private patients. These people, too, have most children. Hence it follows that the "panel" doctor has crowded waiting rooms, long visiting lists, much uncongenial clerical work, many midwifery cases; and much night work. His work generally is so great that it becomes drudgery, and in spite of his best efforts, owing to the great numbers to be attended, it often leads to hurried work and mistakes which could have been avoided had he had more time at his disposal. Time for study there is none. The nature of the panel doctor's work is quite different from that of a hospital physician or surgeon. Most of the ailments he has to treat are trifling, and many of the more serious cases are drafted into hospital and pass from his notice. It cannot be said that this atmosphere or training is one suited to develop the skill required by a hospital physician or surgeon, who must be prepared to investigate patiently, carefully, and thoroughly diseases and conditions requiring great diagnostic ability. Nor is the work of panel doctors likely to lessen in the future, for the number of new medical students in England is diminishing every year, and is only about half what it was thirty years ago. (The figures are:—950 was the average in the twenty years 1880-1899; in 1910 the actual number was 566, and in 1911, 481.) The low number to which it has fallen reflects the uncertainty and instability created in the medical profession by recent legislation.

It is clear that in most provincial hospitals the staffs must come from Class II.

Instead of widening the area from which the staff of a hospital is selected, governors would be acting in the best interests of the patients if they narrowed it, and elected only those men who were willing to practise as physicians or surgeons only. If this were done it would be necessary to diminish their numbers considerably in order that they might make a living as consultants. Men with a good all-round knowledge of their profession develop into the best consultants and specialists. In this case young men with special training from other towns would apply for the posts, and in that sense the area would be widened.

A hospital with members of its staff on the panel will lose caste and prestige and will be degraded, whilst adjoining hospitals, not so staffed, will gain in prestige. Men on the panel naturally object to hospital physicians or surgeons being on the panel too, because, owing to their position, they stand to gain panel patients from them. In addition, a member of a hospital staff could use the hospital as a "dumping" ground for his chronic and troublesome patients, to save himself trouble. Finally, the only argument that can be used by a hospital physician or surgeon wishing to go on the panel is the purely selfish one of remuneration, and not to be entertained for a moment by any committee jealous of the dignity and prestige of their hospital and anxious to do their best for the patients. If it be said that a hospital physician gains little from his appointment, then it will be equally true that he will lose little by resigning it. And if this statement be correct, sooner or later the question will be asked—Should not the staff of hospitals be paid? From every point of view, therefore, a hospital physician or surgeon should be debarred from undertaking work under the Insurance Act. To their assistants, in some institutions, such work may be thought to be allowable.

